# Cerebral schistosomiasis in a patient travelling from São Tomé and Príncipe

**DOI:** 10.1259/bjrcr.20190055

**Published:** 2020-02-12

**Authors:** Rui Duarte Armindo, Sónia Costa, Vânia Almeida, Cândida Barroso

**Affiliations:** 1 Neurology Unit, Hospital Vila Franca Xira, Vila Franca de Xira, Portugal; 2 Neuroradiology Department, Hospital Beatriz Ângelo, Loures, Portugal

## Abstract

Aiming to raise awareness for the possibility of schistosomal involvement of the central nervous system in travellers returning from endemic areas and/or immigrants to nonendemic areas, the authors report a case of neuroschistosomiasis in a Portuguese patient coming from the Republic of São Tomé and Príncipe with good clinical outcome following praziquantel therapy. This is the first case of neuroschistosomiasis associated with São Tomé and Príncipe reported in literature and further studies are needed to confirm which species of this parasite are endemic of that region. We conclude that early diagnosis is key to reduce clinical severity and therefore validation of new diagnostic techniques and establishment of consensual treatment guidelines would be important.

## Clinical presentation

A 35-year-old Caucasian male was admitted to the hospital with severe headaches, focal seizures and mental confusion, 3 weeks after coming back from São Tomé and Príncipe (STP), where he had been living for the past 5 years. His medical history was unremarkable. The patient revealed that 2 weeks before his departure from STP, he had entered a local river against native advice. A few days later he developed a bilateral pruritic rash in the distal half of his lower limbs.

At hospital admission, his general physical examination was normal. Neurologic examination revealed an aggressive behaviour without focal signs or cognitive deficits.

## Investigations/IMAGING FINDINGS

**Figure 1.**
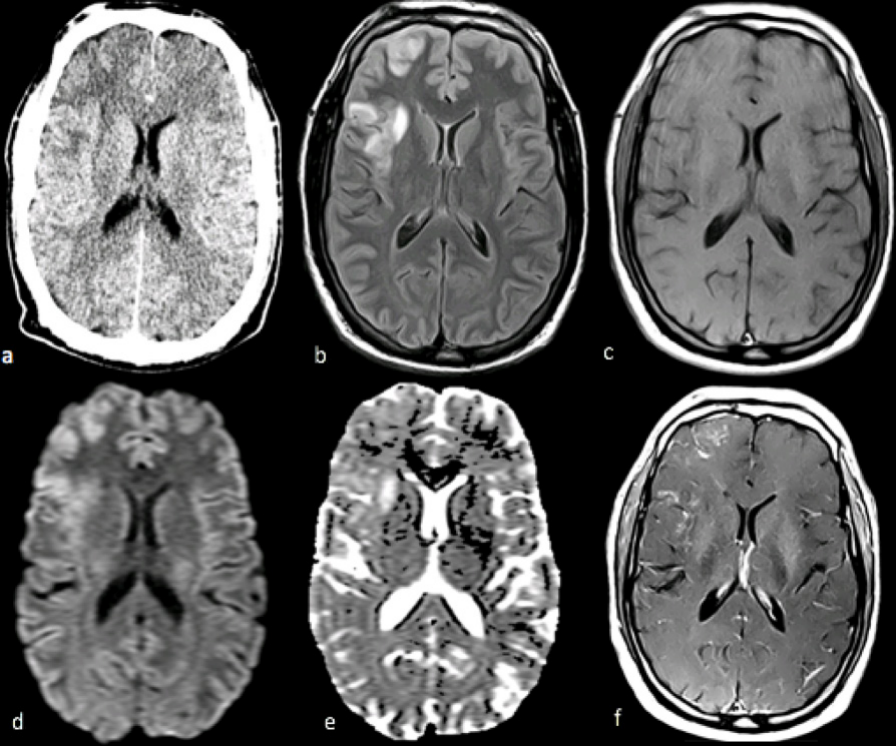
Head NCCT (a) and MRI (b – f) showing hypodensity and mild mass effect on NCCT (a), high signal on FLAIR (b) and patchy *T*1W gadolinium enhancement (c, f) in the cortex and subcortical white matter of the right anterior opercular frontal and insular regions, with additional involvement of the ipsilateral anterior frontal convexity, without restriction on DWI/ADC (d, e). ADC, apparent diffusion coefficient; DWI, diffusion-weighted imaging; FLAIR, fluid-attenuated inversion-recovery; NCCT, noncontrast head CT.

Laboratory findings showed persistent eosinophilia without leucocytosis. Kidney and liver function tests were normal. *Plasmodium* spp was excluded, HIV infection was ruled out and the venereal disease research laboratory test was negative, but serologic tests revealed high titers of anti-*Schistosoma* antibodies (dilution 1/1280; this serologic test detects antibodies for *Schistosoma mansoni*, *Schistosoma haematobium* and *Schistosoma intercalatum*). There was no evidence of parasitic eggs or blood in the urine or stool. The CSF revealed 28 mononuclear cells with normal levels of proteins and glucose. Initial brain imaging findings are reported in Figure 1.

## Differential diagnosis

Clinical presentation and the lesions described by the cranial MRI studies suggested one of the following aetiologies:

- Neoplastic: the images available were not clearly indicative of a space occupying lesion and the relatively fast (over 2 weeks) alterations in size and borders of the lesion did not favour this hypothesis. The uneven pattern of contrast enhancement was also against the typical features of central nervous system (CNS) neoplasm.
- Parasitic: cerebral malaria was put into account early in the diagnostic process since STP is an endemic country for the disease and the clinical presentation was compatible, but the radiological findings were not suggestive of this hypothesis: there was no evidence of recent ischaemic lesions or perivascular haemorrhages. The presence of persistent eosinophilia lead to the request of a larger panel of serologic studies in which antibodies against *Schistosoma* were found. Neurocysticercosis could also be considered but both serological and radiological findings were against it, namely for the absence of cysts around in the areas of documented MR signal change.
- Vascular: CNS vasculitis is a heterogeneous group which could present with variable brain imaging findings. Against this diagnosis were the absence of systemic signs of vascular inflammation and of laboratory inflammatory parameters (normal sedimentation rate, negative ANCA values);
- Others: limbic encephalitis was considered but the topography of the lesion would be very atypical and cerebrospinal fluid analysis was negative for herpes virus. Autoimmune encephalitis could not be excluded but no specific signs pointed towards that possibility. Seizure-related toxicity and other metabolic disorders (*e.g.* hyperglycemic hyperosmolar state) could have similar clinical presentations but the presence of contrast enhancement virtually excluded these diagnoses.

## Treatment

The patient started anticonvulsant therapy (phenytoin 300 mg per day) with good seizure control. Considering the probable diagnosis of cerebral schistosomiasis, the patient started intravenous corticotherapy (methylprednisolone 1g per day, for 5 days) followed by oral praziquantel (40 mg/kg divided in two daily doses, for 3 days) and a switch to oral prednisolone (1 mg/kg/day for 1 month and a tapering off scheme afterwards).

## Outcome and follow-up

**Figure 2.**
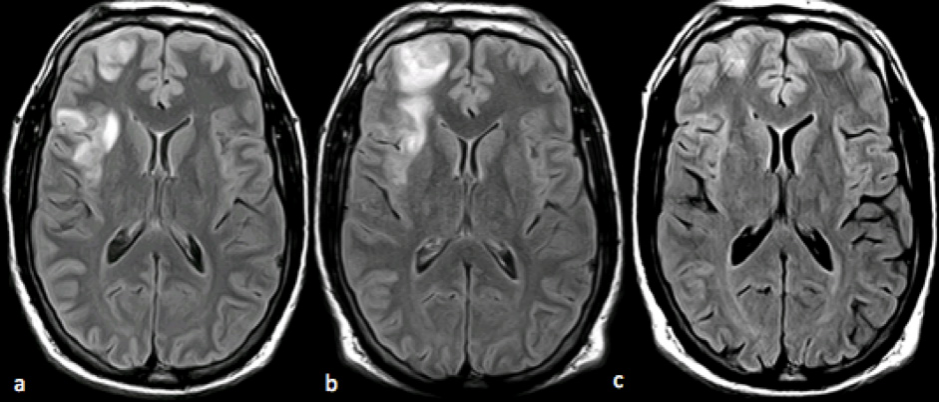
Head MRI axial FLAIR; before treatment (initial MRI, (a); 15 days later, but before initiation of specific treatment (b): showing lesions increased in size with deep white matter involvement and increased mass effect; three months after treatment (c), showing very small residual foci. FLAIR, fluid-attenuated inversion-recovery.

The evolution of the brain imaging findings is shown on Figure 2. There was clinical improvement of headaches and no further convulsive episodes were described, even after the patient decided by his own initiative to stop treatment with antiepileptic drugs (around 6 months after praziquantel treatment).

## Discussion

Schistosomal involvement of the CNS (neuroschistosomiasis) is a condition rarely diagnosed in Europe which can have serious consequences if not treated early.^1^ Portugal is a country historically and geographically related with some African regions where the prevalence of schistosomiasis is high^2^ but reports of cerebral involvement by this parasite are rare. To the best of our knowledge, this is the first case of neuroschistosomiasis associated with the Republic of STP reported in literature.

Schistosomiasis is one of the most widespread parasitic infections in the world,^3^ and it is an important public health problem, mainly in tropical areas.

Neuroschistosomiasis is a rare and severe disorder in which prognosis depends largely on the early establishment of adequate treatment. Almost all reported cases of neuroschistosomiasis are caused by infection with *Schistosoma mansoni*, *Schistosoma haematobium*, or *Schistosoma japonicum*. The first two species, mainly endemic in Africa and South America usually affect the spinal cord. The latter, mostly endemic in Asia, causes more frequently encephalic disease.^4^

According to the World Health Organization, there are no reports indicating the presence of any of the aforementioned *Schistosoma* species in STP. *Schistosoma intercalatum*, the only endemic species of this country,^5^ has never been associated with neurologic involvement of the infection. The lack of specificity of the serologic test used does not permit a final conclusion on this matter. There are also reports of false-positive results in serological assays of patients with cestode infections,^6^ but in this case specific serological assays were negative for neurocysticercosis.

This case presented several diagnostic challenges: there was no evidence of parasitic egg in stool or urine and no brain biopsy was made to allow histopathological confirmation of the parasite’s presence in the patient’s brain.

However, the presence of persisting eosinophilia, a favourable epidemiological context (including the description of the likely moment of infection in a river) and description of an early rash in lower limbs all pointed towards this diagnosis, supported by the clinical and imaging improvement with early and adequate treatment.^7^ This is probably due to acute toxaemic schistosomiasis, a systemic hypersensitivity reaction against the migrating parasite and early oviposition, which usually occurs within 1–3 months of infection.^8^

Many radiological findings can be associated with neuroschistosomiasis, with some studies identifying different types of CNS involvement.^3^ MR findings in this case are consistent with a “pseudotumoural form” previously described in multiple case reports and reviewed by Braga et al,^9^ with irregular contrast-enhancement foci frequently found at the grey matter-white matter junction and an area of perilesional oedema. Even though some authors consider nodular or linear arborizing patterns of enhancement to be characteristic of this condition,^10,11^ neither was evident in this case and other studies identify inflammatory lesions similar to those reported above.^12^ Location at the frontal lobe and existence of a small number of lesions (one or two) are also common aspects at presentation.^10^

Physicians should be aware of this condition in nonedemic areas in travellers returning from endemic areas and/or migrants. As a possibly treatable disease, early diagnosis is needed in order to reduce severity and associated disability levels. It would be important to develop and validate new diagnostic techniques and establish consensual treatment guidelines.

## Learning points

1. Neuroschistosomiasis is a rare disorder but should be considered as a differential diagnosis in endemic areas and patients travelling from endemic areas.
2. Patients may present with neurological involvement even when typical systemic findings (such as urinary or hepatic) are absent.
3. Neuroradiological findings may be variable. Patients may demonstrate a “pseudotumoural form” on MRI.
